# MicroRNA-Target Network Inference and Local Network Enrichment Analysis Identify Two microRNA Clusters with Distinct Functions in Head and Neck Squamous Cell Carcinoma

**DOI:** 10.3390/ijms161226230

**Published:** 2015-12-18

**Authors:** Steffen Sass, Adriana Pitea, Kristian Unger, Julia Hess, Nikola S. Mueller, Fabian J. Theis

**Affiliations:** 1 Institute of Computational Biology, Helmholtz Zentrum München, Ingolstädter Landstr. 1, 85764 Neuherberg, Germany; steffen.sass@helmholtz-muenchen.de (S.S.); adriana.pitea@helmholtz-muenchen.de (A.P.); 2 Research Unit Radiation Cytogenetics, Helmholtz Zentrum München, Ingolstädter Landstr. 1, 85764 Neuherberg, Germany; unger@helmholtz-muenchen.de (K.U.); julia.hess@helmholtz-muenchen.de (J.H.); 3 Clinical Cooperation Group “Personalized Radiotherapy in Head and Neck Cancer”, Helmholtz Zentrum München, Ingolstädter Landstr. 1, 85764 Neuherberg, Germany; 4 Department of Mathematics, Technical University of Munich, Boltzmannstr. 3, 85748 Garching, Germany

**Keywords:** miRNA expression, mRNA expression, mi-/mRNA regulatory network, elastic net regression, local enrichment analysis, head and neck squamous cell carcinoma

## Abstract

MicroRNAs represent ~22 nt long endogenous small RNA molecules that have been experimentally shown to regulate gene expression post-transcriptionally. One main interest in miRNA research is the investigation of their functional roles, which can typically be accomplished by identification of mi-/mRNA interactions and functional annotation of target gene sets. We here present a novel method “miRlastic”, which infers miRNA-target interactions using transcriptomic data as well as prior knowledge and performs functional annotation of target genes by exploiting the local structure of the inferred network. For the network inference, we applied linear regression modeling with elastic net regularization on matched microRNA and messenger RNA expression profiling data to perform feature selection on prior knowledge from sequence-based target prediction resources. The novelty of miRlastic inference originates in predicting data-driven intra-transcriptome regulatory relationships through feature selection. With synthetic data, we showed that miRlastic outperformed commonly used methods and was suitable even for low sample sizes. To gain insight into the functional role of miRNAs and to determine joint functional properties of miRNA clusters, we introduced a local enrichment analysis procedure. The principle of this procedure lies in identifying regions of high functional similarity by evaluating the shortest paths between genes in the network. We can finally assign functional roles to the miRNAs by taking their regulatory relationships into account. We thoroughly evaluated miRlastic on a cohort of head and neck cancer (HNSCC) patients provided by The Cancer Genome Atlas. We inferred an mi-/mRNA regulatory network for human papilloma virus (HPV)-associated miRNAs in HNSCC. The resulting network best enriched for experimentally validated miRNA-target interaction, when compared to common methods. Finally, the local enrichment step identified two functional clusters of miRNAs that were predicted to mediate HPV-associated dysregulation in HNSCC. Our novel approach was able to characterize distinct pathway regulations from matched miRNA and mRNA data. An R package of miRlastic was made available through: http://icb.helmholtz-muenchen.de/mirlastic.

## 1. Introduction

MicroRNAs (miRNAs) represent small-single stranded RNA molecules that own the ability to bind to the complementary 3${}^{\prime }$-untranslated region (3${}^{\prime }$ UTR) of messenger RNA (mRNA) sequences, to post-transcriptionally fine-tune target mRNA gene expression [1,2]. MiRNA biogenesis was proven to be under tight spatio-temporal control and targeting relationships were shown to be largely restricted to specific cell types or tissues [3,4,5]. MiRNAs are thought to act in a combinatorial manner and were also shown to modulate oncogene expression [6,7]. Dysregulation of miRNAs has been associated with human cancer and proven to be sufficient for driving oncogenesis in mouse models, while changes on the genetic and epigenetic levels of the miRNA biogenesis have been associated with cancer initiation [8].

miRNAs may act as oncogenes or tumor-suppressor genes, as demonstrated in various studies across many cancer types [9,10,11], including our own work on head and neck squamous cell carcinoma (HNSCC) [12,13]. HNSCC represents one of the most commonly diagnosed carcinomas worldwide with an incidence of ~600,000 patients per year [14] and is characterized by phenotypic, etiological, biological and clinical heterogeneity [15,16,17,18]. Heavy smoking, alcohol abuse and infection with high-risk types of human papilloma virus (HPV), mostly HPV-16, represent the major risk factors associated with HNSCC. Combined treatment procedures include surgery, radiotherapy alone or in conjunction with chemotherapy or immunotherapy. However, the survival rate for patients with advanced HNSCC remains limited to ~50% [19,20,21]. An oncogenic HPV infection was associated with expression of the viral oncogenes E6 and E7, leading to cell cycle deregulation through E6-induced degradation of p53 and E7-mediated inactivation of the Retinoblastoma (Rb) protein [22,23]. The subsequently caused promotion of cell cycle progression and proliferation were considered to be the onset of HPV-mediated carcinogenesis. HPV+ tumors represent a distinct group within HNSCCs, differing from HPV-tumors in pathogenesis, histopathology, clinical outcome—prognosis was more favorable for HPV+ patients—and molecular biology [15,17,24]. Specific molecular characteristics related to HPV status include gene mutations, genomic copy number aberrations, changes in DNA methylation, mRNA and miRNA expression patterns [17,25]. Lajer *et al.* identified a set of core miRNAs implicated in HPV pathogenesis [25,26]. Strikingly, those HPV core miRNAs are related to the E6/p53 and E7/Rb pathways of HPV induced malignant pathogenesis. The reported involvement of miRNAs in HPV related pathogenesis motivated us to further explore miRNAs associated with HPV infection in HNSCC and to reveal how those miRNAs regulate underlying cellular mechanisms in HNSCCs with respect to HPV status.

Regulation of cellular processes by miRNAs was thought to be mediated through their target genes. It is thus worthwhile to infer target relationships of miRNAs in order to reveal their functional roles. The idea builds on two subsequent steps: (1) identification of mi-/mRNA gene interactions and (2) functional annotation of target genes. As for (1) obtaining mi-/mRNA gene interactions, both computational and experimental methods were used. Several bioinformatics algorithms have been proposed to predict miRNA targets: TargetScan uses a statistical model based on stringent seed pairing, site features and likelihood of preferential conservation [27], miRanda uses a moderately stringent seed pairing algorithm that also considers site number, conservation and free energy [28], while mirSVR represents a machine learning method based on a down-regulation score [29]. The disadvantages of *in silico* target prediction methods reside in the high false positive prediction rates and unspecificity for a given setup [30]. Other approaches inferred miRNA targets solely from expression values [31]. Experimental identification of mi-/mRNA interactions can be achieved through genetic screening, quantification of gene expression changes caused by miRNA transfection or methods based on crosslinking of mRNA and miRNA-containg argonaute (AGO) complexes followed by immunoprecipitation like Photoactivatable-Ribonucleoside-Enhanced Crosslinking and Immunoprecipitation (PAR-CLIP) [32] or High-throughput sequencing of RNA isolated by crosslinking immunoprecipitation (HITS-CLIP) [33]. The StarBase database stores mi-/mRNA target relationships that were identified using PAR-CLIP or HITS-CLIP [34]. Other methods such as the micro-multivariate Markov modeling inference engine (microMUMMIE) [32], PAR-CLIP miRNA assignment (PARma) [35] or MIRZA [36] used CLIP data to computationally infer the specific miRNA that guides the interaction of AGO with a gene target.

While simultaneous measurements of entire transcriptomes including mRNAs and miRNAs have become relatively straightforward with high-throughput techniques, their integration is not trivial. Several methods have been proposed to integrate miRNA and mRNA data for the identification of mi-/mRNA networks. An intuitive approach for finding associations between miRNAs and mRNAs on expression level is correlation analysis [37,38]. Generally, correlation analyses cannot adequately model the demonstrated joint effects of several miRNA on a shared target [39]. To account for the miRNA joint effects, multiple linear regression models have been proposed. An appropriate model, which has been used for target inference on expression level [40], was given by the least absolute shrinkage and selection operator (lasso) regression [41]. It imposes sparsity by an L1 penalty on the regression coefficients shrinking them towards zero, thus allowing for feature selection on the variables in the model. However, lasso selects one representative from each correlated group of miRNAs, so it does not account for co-expression. In contrast to lasso, the ridge regression model maintains all predictors in the model by using an L2 penalty but does, in turn, not perform feature selection. To overcome these drawbacks, an elastic net penalty [42] was used for the integration of miRNA and gene expression data [43], which combines the L1 and L2 penalties in order to account for co-expression among miRNAs and, at the same time, performs feature selection for potential regulatory relationships with target genes. We also previously showed that the usage of elastic net regression in combination with a negativity constraint on the coefficients provides reasonable results both on mRNA and proteome level [44,45].

To reveal functional properties of target gene sets, common approaches were based on statistical enrichment testing to identify over-represented functional categories [46,47,48]. For example, miRGator [47] was proposed to infer miRNA functions by performing a statistical enrichment test of target genes in each term for gene ontology, pathway and disease annotations available on the PhenoMir platform [49]. These methods typically generate a large amount of significantly enriched functional categories, however, many of which are unrelated to the diseases and conditions. A recent study showed that the most commonly used functional enrichment test returns significant *p*-values for targets of randomly selected miRNAs, revealing the intrinsic bias in the target prediction [50]. A combined approach that identifies functional roles of miRNAs from experimental data, together with adequate statistical modeling and miRNA-tailored functional annotation analysis, remains to be done.

**Figure 1:**
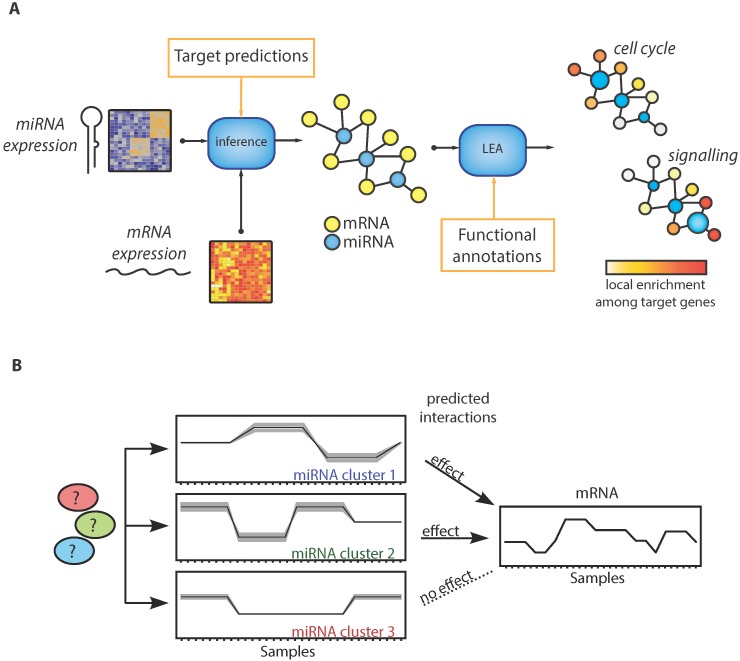
Functional characterization of miRNAs in a condition-specific manner by data-driven inference of mi-/mRNA networks and subsequent functional annotation in the networks. (**A**) MiRlastic uses a two-step approach integrating prior knowledge and mi-/mRNA expressions to infer mi-/mRNA network and miRNA functional annotation; (**B**) Schematic drawing of miRNAs co-expressed in clusters induced by an yet unknown regulatory layer. Only several of the putative miRNA regulators are collectively regulating the mRNA expression.

We have set out to develop a method, called miRlastic, to infer miRNA functions in a data-driven manner using statistical inference of mi-/mRNA interactions, as well as functional characterization of the inferred network (Figure 1A).

To obtain condition-specific mi-/mRNA interactions, we developed a statistical inference method taking into account matched miRNA and mRNA expression data of the underlying conditions together with prior knowledge of sequence-based predictions.

We used a multiple linear regression model with elastic net penalty, which is a trade-off between lasso and ridge regression that accounts for both joint effects of several miRNAs on a common target (Figure 1B) and co-expression between miRNAs [42]. We ensured selecting down-regulation effects with a negativity-constraint on the regression coefficients.

On synthetic data, miRlastic outperformed commonly used methods and was especially suitable for low sample sizes. To functionally annotate miRNAs based on the inferred mi-/mRNA network, we introduced a local enrichment analysis that scores miRNAs given the underlying network structure with respect to functional annotations of target genes.

We thoroughly evaluated miRlastic on a cohort of HNSCC patients provided by The Cancer Genome Atlas (TCGA). We inferred an mi-/mRNA regulatory network for human papilloma virus (HPV)-associated miRNAs in HNSCC. The resulting network best enriched for experimentally validated miRNA-target interactions, when compared to commonly used methods. Finally, the local enrichment analysis (LEA) procedure identified two functionally distinct clusters of miRNAs that were predicted to mediate HPV-associated dysregulation in HNSCC. An R package of miRlastic was made available through miRlastic [51].

## 2. Methods and Materials

### 2.1. HNSCC Data

We downloaded Level 3 mRNA and miRNA RNA-seq data from the TCGA data portal. The mRNA Level 3 data consisted of gene expression measurements which were generated following the protocol previously described by the TCGA consortium [52]. For analysis on mRNA expression level, we selected only the mRNAs with non-zero count values in more than 80% of the patients, non-zero standard deviation and applied a log2 transformation. For miRNA analysis, we obtained normalized expression levels for miRNA precursors after selecting only those miRNAs that accomplished the same criteria as the mRNAs. In the first step, we overlaid the precursor entries with associated entries in TargetScan. By doing so, we considered only those miRNAs that are supposed to be incorporated into the RNA-induced silencing complex (RISC) complex and thus not subject to degradation [53]. For each miRNA, for which TargetScan provides predictions on both -3p and -5p strands, we unify the two sets of putative targets and assign them to the corresponding precursors. After running the inference step, we intersect the resulting mi-/mRNA interactions from the individual precursors with the predictions of the mature miRNAs provided by TargetScan. We performed differential miRNA expression analysis on a subcohort of 244 patients for which the human papillomavirus (HPV) status clinical parameter was provided [17] in order to identify deregulated miRNAs between HPV+ and HPV- patients. When using the edgeR package [54], we also included the age and gender as confounder variables. We controlled for a 5% false discovery rate (FDR) using Benjamini and Hochberg algorithm [55].

### 2.2. Preliminaries

We defined ***X*** and ***Y*** as two matrices that contain miRNA $\left(x_{jk}\right)$ and mRNA $\left(y_{ik}\right)$ expression data, both simultaneously measured in *s* samples, with k∈{1,...,s}, i∈{1,...,n}, j∈{1,...,m}, such that *n* and *m* represent the number of measured miRNAs and mRNAs, respectively. We denoted the regulatory interaction of miRNAs and their putative mRNA targets as a bipartite graph *G*. The bipartite graph *G* captures all putative mi-/mRNA interactions in $G=(V^{miR},V^{mR},E)$ with disjoint sets of two node types as $V^{miR}$ and $V^{mR}$. The set of all mRNAs represents all the nodes listed in $V^{mR}=\left\{v_{1}^{mR},...,v_{m}^{mR}\right\}$, and likewise the miRNAs represent the nodes in $V^{miR}=\left\{v_{1}^{miR},...,v_{n}^{miR}\right\}$. The edge set $E=\{e_{1},...,e_{z}\}$ connects the nodes from $V^{miR}$ with the ones from $V^{mR}$ as $e_{l}=\left(v_{u}^{miR},v_{w}^{mR}\right)$ with l∈{1,...,z}. The edges $e_{l}$ were extracted from the TargetScan database (Version 6.2) [27] and used as input graph *G* for the miRlastic inference method yielding an optimized graph, $G^{\prime }=\left(V^{miR},V^{mR},E^{\prime }\right)$ with $E^{\prime }\subseteq E$.

### 2.3. Group-Wise Correlation of miRNAs with a Shared Target Gene

**Figure 2:**
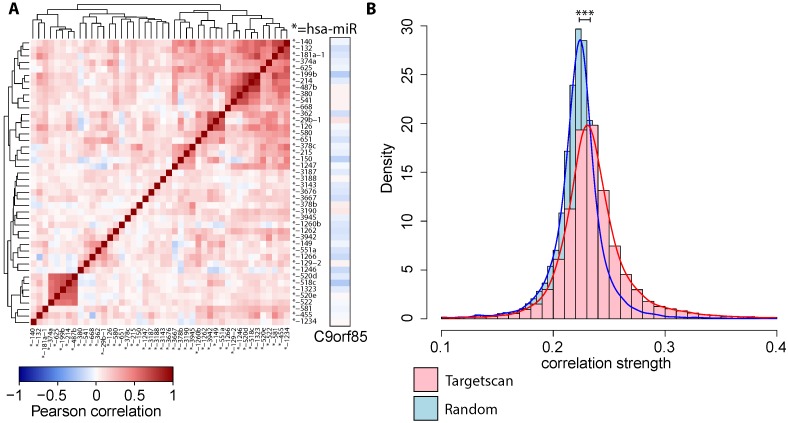
Collective effects of co-expressed miRNAs. (**A**) Pairwise correlation of putative, expressed miRNA regulators together with their target gene C9orf85 (obtained from the HNSCC dataset). The miRNAs are themselves clustered into several co-expressed groups; (**B**) The distribution of correlation strengths c(X) of miRNA sets, which are predicted to target a common gene, (red curve and histogram) is higher than for randomly re-sampled mi-/mRNA associations (blue, Wilcoxon rank sum test has $p<1\times 10^{-80}$) in the HNSCC miRNA expression dataset.

We exemplary calculated pairwise Pearson correlation coefficients among all miRNA expression profiles from the HNSCC dataset, which were predicted by TargetScan with the common target C9orf85 (Figure 2A). Note that this data was representative for the entire dataset. We observed subgroups of high correlation which corresponds to the expectation that functionally related miRNAs or miRNAs in high proximity on the chromosome tend to be co-expressed [7,56].

To systematically analyze whether miRNA expression profiles were typically correlated when sharing a putative target, we next defined a measure of correlation strength c(X) for a matrix of miRNA expression measurements $X=(x_{ik})$ as

$$c\left(X\right)=\frac{{\left||R\left(X\right)|\right|}_{F}}{\sqrt{(n^{2}-n)/2}}$$

with the Pearson correlation matrix $R\left(X\right)=\left(\rho_{i_{1}i_{2}}\right)=\left(r_{X_{i_{1}\cdot }X_{i_{2}\cdot }}\right)$ for $i_{1},i_{2}\in i$. The Frobenius norm of the correlation matrix R(X) was calculated as

$${\left||R\left(X\right)|\right|}_{F}=\sqrt{\sum_{i_{1}<i_{2}}\rho_{i_{1}i_{2}}^{2}}$$

Note that only the upper triangular matrix with $(n^{2}-n)/2$ elements was considered for the calculation of the Frobenius norm. As all elements of R(X) range between [-1,1], all values of c(X) range between [0,1]. The extreme values c(X)=0 and c(X)=1 indicate an entirely uncorrelated and perfectly (anti-)correlated set of miRNAs, respectively.

Using the correlation strength measure c(X), we evaluated the properties of collective miRNA regulation by assessing the (anti-)correlation strength across all miRNAs, which are predicted to target a common mRNA. We found that these sets of miRNAs were more correlated among each other than randomly sampled sets of miRNAs ($p<1\times 10^{-80}$ Wilcoxon rank sum test, Figure 2B).

### 2.4. mi-/mRNA Network Inference

To model the regulatory behavior of several miRNAs jointly modulating one common mRNA target $v_{i}^{mR}$, we extracted from *G* all connected $n^{*}$ miRNAs $v_{i^{*}}^{miR}$ with $i^{*}=\left\{i|\exists \left(v_{i}^{miR},v_{j}^{mR}\right)\in E\right\}$. We refer to these observations of one mRNA *j* as $y_{j}$ and to its associated miRNA observations as $X\left(j\right)=X_{i^{*}}^{T}$, where the one-dimensional vector $y_{j}$ and the $s\times n^{*}$-dimensional matrix X(j) represent the response and the predictors of a regression model.

The mi-/mRNA interaction can be modeled without feature selection by a linear regression model, separately for each mRNA as:

$$y_{j}∼\beta_{j0}+X\left(j\right)\beta_{j}+\epsilon$$

with the normally distributed error ϵ∼N(0,σ), parameters $\beta_{j}=\left(\beta_{j1},...,\beta_{jn^{*}}\right)$ and intercept $\beta_{j0}$.

As shown above, high co-expression among miRNAs is typical for miRNAs with functional similarity and thus, needs to be taken into account when performing feature selection. From correlation between miRNAs and their target gene, the effects of joint actions of miRNAs were detectable.

For network inference, we next performed feature selection using penalized regression (elastic net [42]). We therefore introduced a negativity constraint on the coefficients $\beta_{j}$ to account for the fact that miRNAs mainly down-regulate mRNA target expression levels [57]. The $\beta_{j}$ values were obtained by solving the following optimization problem:

$${\hat{\beta }}_{j}=\underset{\beta_{j}}{\mathrm{argmin}}|y_{j}-X\left(j\right)\beta_{j}+\lambda P_{\alpha }\left(\beta_{j}\right)|,$$

where

$$\begin{array}{cc} P_{\alpha }\left(\beta_{j}\right) & =\left(1-\alpha \right)\frac{1}{2}\left|\right|\beta_{j}{\left|\right|}_{2}^{2}+\alpha ||\beta_{j}{\left|\right|}_{1}. \end{array}$$

The parameter $\alpha_{j}$ with $0\leq \alpha_{j}\leq 1$ denotes the elastic net mixing parameter. The second parameter *λ* was identified by 10-fold cross-validation using the glmnet package. For estimating the regression coefficients ${\hat{\beta }}_{j}$, we used a coordinate descent approach [58], which is also implemented in the glmnet package.

To tune the elastic net penalty, we adjusted *α* with respect to the potentially expected fraction of correlated predictor groups. The parameter $\alpha_{j}$ of the elastic net regression model of $y_{j}$, given X(j), is then defined as $\alpha_{j}=10^{-c(X(j))}$.

The choice of the parameter *α* allowed for an unbiased parameter tuning whereas lower values of $\alpha_{j}$ were slightly preferred. However, we wanted $\alpha_{j}$ to remain non-zero in any case such that feature selection was performed in all models. Therefore, gene-specific choice of $\alpha_{j}$ parameter was a good trade-off.

### 2.5. Functional Characterization of miRNA-Target Networks

To identify local, closely-connected functions within the inferred network, we designed a local enrichment analysis (LEA) procedure. We evaluated whether node arrangements were assigned to a certain term, describing e.g., a molecular function or biological process, occurred by chance or not. Proximity of two target gene nodes in the network were measured by their shortest path. The bipartite graph $G^{\prime }=\left(V^{miR},V^{mR},E^{\prime }\right)$ was the input for LEA. The edges and edge weights were represented as a matrix $W=(w_{ij})$ with *z* non-zero entries. In this case, edge weights were given by scaled negative regression coefficients. We transformed weights by $e^{w}$ to obtain positive edge weights.

A path between two gene nodes was defined as $P\left(a,b\right)=\left(v_{1}^{mR},...,v_{p}^{mR}\right)$ with $v_{1}^{mR}=v_{a}^{mR}$ and $v_{p}^{mR}=v_{b}^{mR}$ such that there exists an miRNA node connected to both nodes $v_{k}^{mR}$ and $v_{k+1}^{mR}$ in P(a,b) for all 1≤k<p. The distances of a path between two nodes $v_{a}^{mR}$ and $v_{b}^{mR}$ were computed as:

$$d\left(a,b\right)=\sum_{k=1}^{p-1}\min_{i^{*}}\left(w_{i*k}+w_{i*k+1}\right)$$

where $i^{*}$ denotes the miRNAs that target the mRNAs *k* and k+1. A path P(a,b) between the mRNAs *a* and *b* is then called shortest path $P_{min}\left(a,b\right)$ if it minimizes the distance d(a,b).

Next, we scored local neighborhoods in the network. Let $M_{k}=g_{1k},...,g_{mk}$ denote the gene set consisting of *m* annotated genes for a specific term *k* with k∈1,...,l, which may be, for example, retrieved from an online repository like the Kyoto Encyclopedia of Genes and Genomes (KEGG) [59], and let $M_{k,G^{\prime }}:=M_{k}\cap V^{mR}$ be the set of genes in $M_{k}$ that overlap with the genes in $G^{\prime }$. In order to determine the enrichment of $M_{k,G^{\prime }}$ around a certain mRNA *j* in $G^{\prime }$, we compared the distribution of the shortest path distances $D_{min}\left(j,M_{k,G^{\prime }}\right)$ to the background distribution $D_{min}\left(j,G^{\prime }\backslash M_{k,G^{\prime }}\right)$. Note that $D_{min}\left(j,M_{k,G^{\prime }}\right)$ included the shortest path distance to a node itself, which is defined as zero, if $v_{j}\in M_{k,G^{\prime }}$. We applied a left-tailed Wilcoxon rank-sum test, which resulted in a *p*-value indicating whether the values of $D_{min}\left(j,M_{k,G^{\prime }}\right)$ were significantly shifted towards lower values when compared to values of $D_{min}\left(j,G^{\prime }\backslash M_{k,G^{\prime }}\right)$. We used *p*-values to assess a score $S(v_{j})$ as $S\left(v_{j}\right)=-\log_{10}\left(p_{j}\right)$ that described the enrichment of the term for the given functional group $M_{k}$ around gene *j*.

To finally characterize the importance of miRNAs in $G^{\prime }$, we calculated the miR score $S_{miR}\left(v_{i}\right)$ for every node $v_{i}\in V^{miR}$ by considering the set $V:=\{v_{j}|\left(v_{j}\in V_{mR}\right)\wedge \left(\exists \left(v_{i},v_{j}\right)\in E\right)\}$ of associated mRNA nodes. We also introduced a weight that accounts for the number of corresponding targets $|V_{i}|$ for each miRNA. The score was then defined as:

$$S_{miR}\left(v_{i}\right)=\left(\frac{1}{|V_{i}|}\sum_{v_{j}\in V_{i}}S\left(v_{j}\right)\right)\cdot \sqrt[{}]{|V_{i}|}.$$

**Figure 3:**
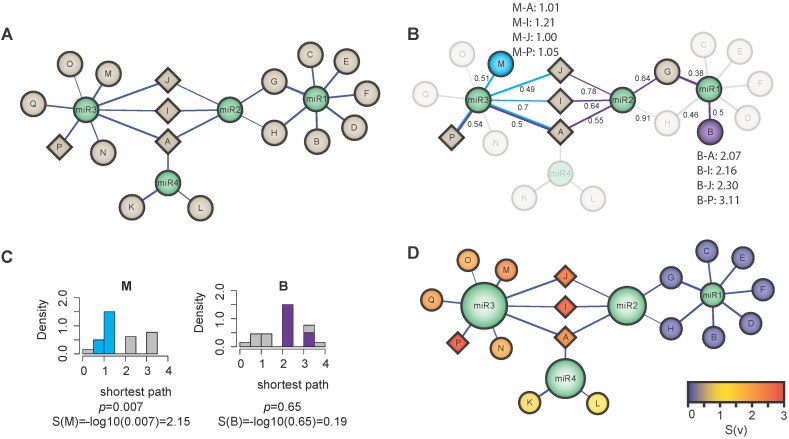
Node scoring strategy of local enrichment analysis. (**A**) Example of a network in which the nodes are represented by miRNAs (green) and genes (grey) and the edges are represented by the mi-/mRNA interactions. The network is given as input for the functional annotation step. We assume that four genes are assigned to a certain functional group—A, I, J, P (diamond shape); (**B**) The transformed network after computing the shortest path distances between these four nodes and the two nodes M (blue) and B (purple). The edge labels denote the weights after the transformation. The edge weight indicates the strength of the mi-/mRNA negative regulation; (**C**) The distribution of shortest paths from node M to the nodes A, I, J and P is significantly shifted to lower values. No shift can be observed for node B. The *p*-values determined by left-tailed Wilcoxon rank-sum test are converted to the node scores; (**D**) Network visualization after annotating functional groups. The node scores are indicated by the color. The size of the miRNA nodes corresponds to the miR score.

Figure 3 illustrates the LEA scoring. The inferred mi-/mRNA interactions were subjected to LEA with the nodes A, I, J, P being part of the gene set assigned to the term (term nodes, Figure 3A, diamond shaped). Exemplary shortest distances between node M (blue) or B (purple) and respective term nodes were calculated.

We next compared the distributions of shortest distances to the background distribution that represents the shortest distances to genes, which did not overlap with the gene set assigned to the term (Figure 3C). We observed that, in the case of node M, the distribution of shortest distances to the gene set assigned to the term tends to be shifted to lower values as compared to the background distribution, which was not the case for node B. In order to statistically test for this shift to lower values, we apply a left-tailed Wilcoxon rank-sum test and obtain a significant *p*-value that is $\left(p=7\times 10^{-3}\right)$ in the case of node M, while the *p*-value for node B was not significant $p=6.5\times 10^{-1}$. We can thus conclude that node M was actually located in close proximity of the nodes associated with the term *k*, whereas node B is not. Given the *p*-values from the left-tailed Wilcoxon rank-sum test, we can calculate the scores of the two nodes M and B as $S\left(M\right)=-\log_{10}\left(7\times 10^{-3}\right)=2.15$ and $S\left(B\right)=-\log_{10}\left(6.5\times 10^{-1}\right)=0.19$, respectively. This score indicated the proximity of a gene to the genes in the functional group.

Finally, we were interested in finding local enrichment of a set of terms given their associated gene sets. With $G^{\prime }$ being the mi-/mRNA network and $M_{k,G^{\prime }}$ the set of overlapping genes between the gene set $M_{k}$ of term *k* and the genes in $G^{\prime }$, we selected all shortest distances $D_{min}\left(M_{k,G^{\prime }}\right)$ between the nodes $v_{j}\in M_{k,G^{\prime }}$. We compared the distribution of this set to the distribution of all other shortest distances $D_{min}\left(V_{mR}\backslash M_{k,G^{\prime }}\right)$. We applied a left-tailed Wilcoxon rank-sum test to assess the enrichment of associated terms in a local area of the network. Terms were then considered to be locally enriched if their adjusted *p*-values (Bonferroni corrected) were below a significance threshold of p<0.05.

### 2.6. Implementation and Availability

The whole miRlastic pipeline was implemented within the R environment for statistical computing [60]. For elastic net regression we used the *glmnet* package [58]. To calculate the shortest paths in a given mi-/mRNA network, we used the implementation of Dijkstra’s algorithm [61] in the igraph [62] package.

MiRlastic can be downloaded as an R package from http://icb.helmholtz-muenchen.de/ mirlastic [51].

### 2.7. Synthetic Data

**Figure 4:**
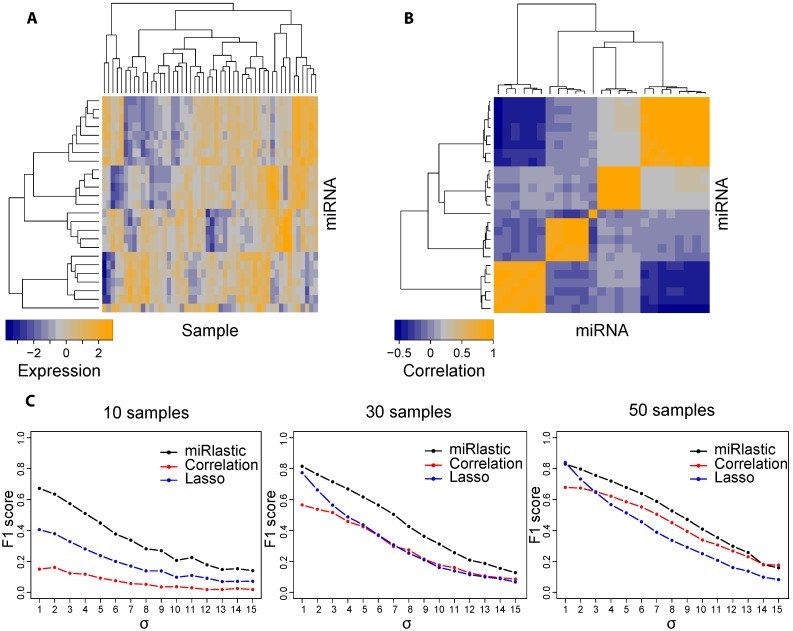
Benchmark with synthetic data. (**A**) Heatmap illustrating a set of randomly generated synthetic miRNAs with 30 samples; (**B**) Heatmap of pairwise correlations between the generated miRNAs; (**C**) Success-rate ($F_{1}$) of all algorithms across varying sample numbers and noise levels to recover the true synthetic mi-/mRNA associations.

We set up a test environment: in each of the test runs, we generated a set of synthetic miRNA and mRNA expression values adapting to biological features. We modeled a set of miRNAs with expression values $x_{i}∼N\left(0,1\right)$ targeting a common mRNA. Furthermore, expression levels were modeled for a set of unknown factors, $h_{j}∼N\left(0,1\right)$ which were assumed to target a distinct subset of the predicted miRNAs (Figure 4a). We generated synthetic miRNA expression values by assuming a randomly coordinated regulation as it is the case, for example, for clustered miRNAs. Assuming a repressive effect of the targeting miRNAs, the mRNA expression profile was then generated as

$$y=\sigma \epsilon +\sum_{i}-{\hat{x}}_{i}$$

where ϵ∈N(0,1) corresponds to the noise arising from biological reasons or experimental artifacts. We performed tests with different magnitudes of the error weight *σ*. In addition to the miRlastic inference, we also applied correlation analysis and lasso on the generated profiles. For the correlation analysis, a synthetic miRNA was considered to be a true regulator of a mRNA if the adjusted *p*-value (Bonferroni corrected) of the negative Pearson correlation coefficient was below 0.05. For lasso, we used the miRlastic inference method with a fixed α=1. The entire procedure was repeated three times with 10, 30 and 50 samples each across 500 runs and varying noise levels. In each parameter setting, the corresponding $F_{1}$ measure was calculated.

We counted the number of falsely chosen miRNAs (false positives, FP) and the number of correctly missed miRNAs (false negatives, FN) obtained after several runs. Based on precision and recall rate, we computed $F_{1}=2\cdot \left(\mathrm{precision}\cdot \mathrm{recall}\right)/\left(\mathrm{precision}+\mathrm{recall}\right)$, where precision=TP/(TP+FP) and recall=TP/TP+FN, for any related confusion matrix comparing actually classes (true and false) to any classification results (positive and negative).

### 2.8. Validation of Network Inference on Experimental HNSCC Data

We compared the network inference results, by accounting for how many mi-/mRNA target predictions were experimentally validated. Experimentally validated interactions were downloaded from StarBase [34]. As background set, we considered only those mi-/mRNA interactions with medium stringency from the starBase database intersected with the set of miRNAs and mRNAs also predicted by TargetScan. Note that TargetScan was used as prior network for miRlastic and all related methods, such that we compared all inference methods to the fraction of experimentally validated interactions in TargetScan itself. For validation, we performed Fisher’s exact test to test whether inferred mi-/mRNA pairs by one method (e.g., inferred using miRlastic) were enriched for experimentally validated pairs from StarBase.

## 3. Results and Discussion

We extensively evaluated the performance of the miRlastic network inference using synthetic data and further applied the miRlastic pipeline on HNSCC patients available on the TCGA data portal.

### 3.1. Robust mi-/mRNA Network Inference for Small Sample Sizes

In order to assess the performance of our inference approach, we built a test environment generating synthetic miRNA and mRNA expression profiles with characteristic biological features. We generated expression profiles of sets of miRNAs targeting the same mRNA (Figure 4A) such that miRNAs were set-wise highly correlated among each other while the correlation to miRNAs in different groups was rather low (Figure 4B). The mRNA expression was then calculated as being regulated by only a subset of miRNAs and additional noise. The set-wise miRNA correlations for the synthetic expression data was in accordance with the HNSCC expression data as shown in Figure 2A.

We evaluated the performance of miRlastic in comparison with the related methods, namely pairwise Pearson correlation analysis and lasso. Correlation analysis showed low performance for low sample numbers, whereas the results improved for high sample numbers. Lasso performed good for medium and high sample numbers, yet only for low noise levels, indicating reduced robustness against noisy observations. Throughout all settings, the miRlastic inference method outperformed correlation analysis and Lasso, especially for low sample numbers (Figure 4C).

The benchmark on synthetic data indicated that the miRlastic inference method was able to provide reasonable results even when applied on low-dimensional datasets. This comes as another advantage, since low sample datasets are ubiquitous in biological research due to the costs required for large-scale techniques. The number of matched samples tend to be lower especially for combined expression data (miRNA and mRNA), since the measurements need to be performed twice. Here, we could show that the miRlastic inference was able to reliably identify true regulators with high specificity and sensitivity in a biologically reasonable synthetic test environment.

### 3.2. mi-/mRNA Regulatory Network of Head and Neck Squamous Cell Carcinoma

**Figure 5:**
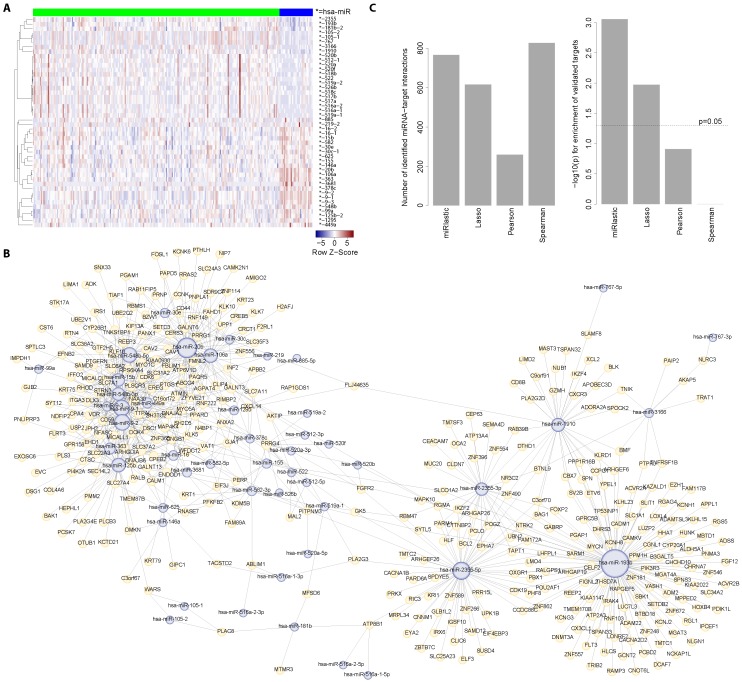
mi-/mRNA regulatory network inference in HNSCC samples. (**A**) 44 miRNA expression profiles show differential expression between HPV+ (blue) and HPV- (green) patients; (**B**) mi-/mRNA regulatory network generated by our inference algorithm. The network consists of 766 regulations between 44 miRNAs (light blue) and 16,617 genes (light yellow). The edges represent miRNA-target relationships in the context of the TCGA HNSCC cohort; (**C**) Performance evaluation of the mi-/mRNA associations inference module. The left bar plot indicates the number of mi-/mRNA interactions detected by our approach, lasso, Pearson and Spearman. The right bar plot shows the -log10-transformed *p*-values when testing for enrichment of experimentally validated targets within the results of each method. We provide an interactive representation of this network at http://icb.helmholtz-muenchen.de/mirlastic/hnscc [71].

To understand HPV-associated miRNA-mediated gene regulation of HNSCC, we applied the miRlastic inference to the HNSCC TCGA data set. As HPV was shown to disrupt cellular differentiation in HNSCC [17,63,64], we used only those 244 patient samples with reported HPV status. Subsequent to preprocessing, target predictions were available for 16,617 mRNAs and 600 miRNAs. We performed a differential analysis between HPV+ and HPV- patients yielding 44 significantly differentially expressed miRNAs (Figure 5A). Among the differentially expressed miRNAs, we observed the miR-9 family, miR-363, miR-20b which have been associated with the HPV status in several independent studies [26,65,66,67,68,69]. In agreement with the results of the Wald *et al.* study [70], miR-363 showed up-regulation in the HPV infected patients. We performed the miRlastic inference using 135,391 targets predicted by TargetScan in combination with the respective miRNA and mRNA expression values. MiRlastic inferred 766 mi-/mRNA interactions (Figure 5B).

We next asked whether the network inferred by miRlastic was specifically enriched for experimentally validated mi-/mRNA interactions. Therefore, we collected experimentally validated interactions from StarBase and compared performance to three other existing methods, namely lasso and Pearson as well as Spearman correlation analysis. A Fisher’s exact test was conducted in order to determine whether the fraction of inferred and validated interactions was higher than expected from the prior target network (TargetScan) with respect to the number of inferred interactions (Figure 5C). For miRlastic, the test yielded a highly significant *p*-value of $p=8.736821\times 10^{-4}$. We obtained a *p*-value of $p=1.059271\times 10^{-2}$ for lasso, $p=1.228527\times 10^{-1}$ for Pearson correlation and $p=9.978787\times 10^{-1}$ for Spearman. These results indicated that the miRlastic approach was able to identify a higher fraction of validated target predictions as compared to the other methods. Lasso also performed well with a significantly fraction of experimentally identified interactions but when compared to miRlastic was clearly lower (Figure 5C). Pearson and Spearman correlation did not show any significant difference. Taken together, miRlastic inference outperformed other commonly used methods with respect to the over-representation of experimentally validated mi-/mRNA interactions.

### 3.3. Two Functional Clusters in miRNA-Mediated HPV-Associated Dysregulation

In the previous sections, we described the network that resulted from the analysis of mi-/mRNA data from tumors of patients from the TCGA HNSCC cohort with known HPV status. To finally gain insights into the functional role of the identified mi-/mRNA pairs, we analyzed miRNA-target genes for local enrichment given the HNSCC network. For the application of LEA on the HNSCC mi-/mRNA interaction network, we downloaded 108 pathways from KEGG for gene annotations [59].

**Figure 6:**
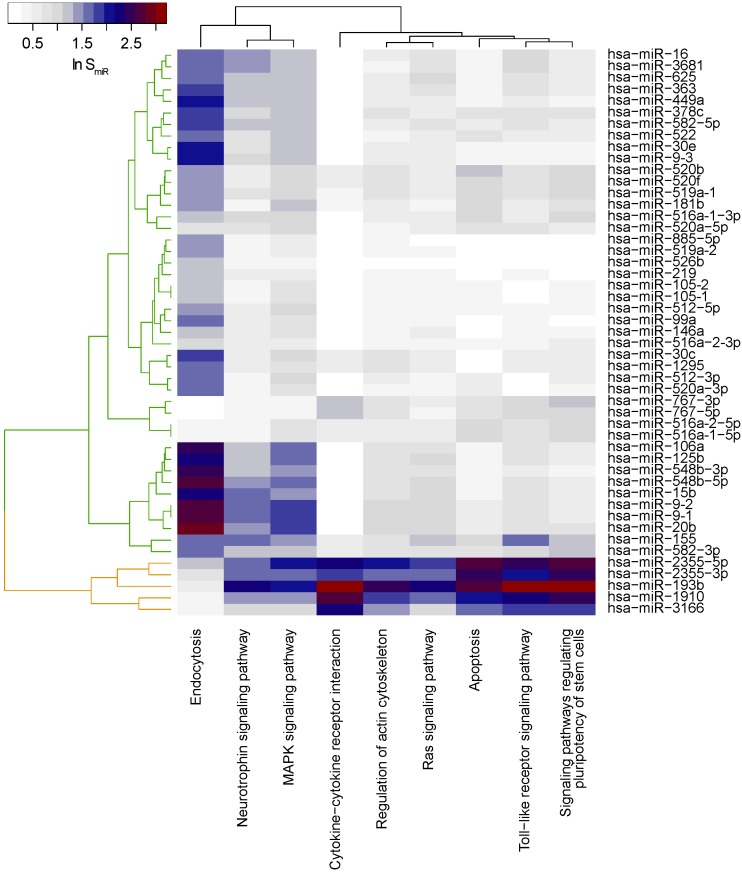
Functional characterization of the HNSCC mi-/mRNA network. Heatmap of miR scores $S_{miR}\left(v\right)$ for each miRNA *v* in the network indicating the functional role in the significantly locally enriched KEGG pathways.

In total, we obtained nine significantly locally enriched pathways (Figure 6). Overall, distinct frequencies of genetic alterations affecting the key signaling pathways of G protein Ras (RAS) and mitogen-activated protein kinases (MAPK), as well as apoptosis, were reported before for HPV+ and HPV–HNSCC on gene level: for example, TP53, HRAS, MYC, BIRC2 and CASP8 were most often altered in HPV- tumors, whereas HPV+ cases were characterized by PIK3CA mutations, inactivation of TRAF3, the viral genes E6, E7 and amplification of E2F1 [17].

We next clustered miRNAs according to their functional miR score that reflected the strength of interactions of miRNAs with a particular pathway identified two clusters with similar pattern of miR scores across pathways (Figure 6).

The two pathway MAPK- and neurotrophin-signaling pathway associated with both clusters suggesting that they were orchestrated by most miRNAs in HPV-associated dysregulation. Notably, MAPK-, RAS-, neurotrophin and toll-like receptor (TLR) signaling share common elements such as a series of MAPKs, protein kinase B (AKT) and most importantly the downstream located transcription factor NF-*κ*B, which is involved in regulating chief processes governing tumorigenesis such as immune response, apoptosis, proliferation and angiogenesis [72,73]. Interestingly, hsa-miR-193b showed a strong association with these pathways which was so far shown to play important roles in MAPK signaling [74] and independently shown for HNSCC [75]. This finding validated the miRlastic approach analyzing miRNA regulation in a data-driven manner with subsequent scoring by functional annotation in a network-context.

The first miRNA cluster was characterized by associations of many miRNAs (hsa-miR-106a, -125b, -548b-3p/5p, -15b, -9-2, -9-1, -20b, -155, and -582-3p) mostly with the endocytosis pathway. Endocytosis was shown to be deregulated in cancer cells in such a way that cell surface molecules with growth advantage effects were recycled at higher rates while molecules with reduced growth effects or those making cells recognized by the immune system were removed from the cell surface [76].

The second miRNA cluster was functionally broader and characterized by associations between hsa-miR-2355-5p/3p, -193b, -1910 and 3166 with cytokine-cytokine receptor (CCR) interaction, regulation of actin cytoskeleton, RAS signaling, apoptosis, toll-like receptor (TLR) signaling and signaling involved in regulating pluripotency of stem cells. Disruption of the apoptosis pathway is one of the hallmarks of cancer and is the result of bypassing cell cycle control in cancer cells, which, in turn, leads to uncontrolled proliferation of cells [77]. Disturbance of the regulation of the actin cytoskeleton was shown to play a role in the motility and invasion potential of HNSCC cells, which were key features of metastasizing cells [78]. Cancer stem cells (CSC) were described to be involved in therapy resistance of HNSCC, while HPV+ HNSCCs have been shown to harbor smaller proportions of CSC compared to HPV–HNSCCs [79]. Consequently, these results provided a possible explanation for the favorable prognosis of HPV+ HNSCC patients and link the pathway “signaling pathways regulating pluripotency of stem cells” also to HPV-infection. The expression patterns of TLR, which not only play a role in the immunological defense against pathogens but also in cancer, were demonstrated to be related to the HPV-status [80]. The HPV oncogenes E6 and E7 have been shown to reduce expression of TLRs, thus linking the TLR signaling pathway to HPV+ HNSCCs [81].

The oncoproteins E6 and E7 are known to bind several cellular targets, which have been described to regulate, among others, cytokine signaling, thereby providing a link between HPV-status and the “cytokine-cytokine receptor interaction” pathway [82]. Cytokine-cytokine receptor interaction in general is a common mechanism of cell-cell communication between tumor cells and surrounding non-tumor cells and plays an important role in regulation of the tumor driving mechanisms immune suppression and angiogenesis [83]. The miRlastic analysis suggested that few miRNAs, predominantly hsa-miR-193b and -2355-5p, mediated gene dysregulation largely through modulation of TLR–CCR-interaction signaling and signaling regulating pluripotency of stem cells.

In all, the pathways identified applying miRlastic were in context with signaling disturbance known to be associated with HNSCC tumorigenesis. Moreover, the identified pathways were also known to be related to HPV-status of HNSCC, strengthening the validity of the generated mi-/mRNA regulatory network. Our finding suggested that the two clusters of miRNA-mediated target gene regulation were characterized by distinct signaling pathways deregulated in HPV+ *versus* HPV-HNSCC and, therefore, support plausibility of the results in the context of the biology of HPV+ HNSCC tumors.

## 4. Conclusions

We have demonstrated the power of coupling an miRNA-characteristic mi-/mRNA target inference method to a local enrichment analysis on the inferred network, in order to identify functional roles of miRNAs in a given experimental setting. The proposed target inference of miRlastic employed a linear regression model with elastic net penalization and negativity-constrained coefficient estimation. The choice of balancing between L1 and L2 was achieved by capturing the co-expression structure of miRNAs independently for each common target gene. The miRlastic inference was able to best enrich for experimentally validated interactions. The local enrichment analysis and subsequent clustering of functional miR scores across pathways, finally allowed us to assign functional roles to sets of miRNAs as given by short distances in the inferred networks between target genes of a selected pathway. The method was made publicly available as an R package. We applied mirRlastic to HPV-associated mi-/mRNA regulation in HNSCC data and were able to identify two miRNA clusters of each distinct functional roles. The HPV-associated analysis in HNSCC patients provided, via the identified mRNA targets, descriptive and mechanistic insights into the molecular phenotype of HPV-driven HNSCC and further validated the meaningfulness of the miRlastic method at a biological and clinical level. We used precursor miRNA expression instead of mature miRNA expression, which was used in previous applications of our approach [44,45], and thus proved that miRlastic is independent from the transcriptional status. In summary, we designed a method for which we showed that it provided a valuable basis for identifying functional roles of miRNAs in a disease context. On the one hand, we selected miRNAs that have a promising role as potential biomarkers due to the significant association with a disease-specific cellular process. On the other hand, this knowledge can further be used to identify potential therapeutic targets, e.g., for cancer treatment, since it has been shown that tumor growth may be prevented either by systemic administration of miRNAs [84] or miRNA silencing [85].
